# More than meets the eye: The hidden burden of temporary feeding tubes on children and their families

**DOI:** 10.1002/ncp.70048

**Published:** 2025-10-26

**Authors:** Claire Reilly, Jasmine Foley, Rebecca Packer, Nikhil Thapar, Syed Afroz Keramat, Jeanne Marshall

**Affiliations:** ^1^ School of Health & Rehabilitation Sciences, The University of Queensland Brisbane Queensland Australia; ^2^ Queensland Children's Hospital, Children's Health Queensland Hospital and Health Service South Brisbane Queensland Australia; ^3^ Gastroenterology, Hepatology and Liver Transplant, Queensland Children's Hospital Brisbane Queensland Australia; ^4^ Faculty of Health, School of Exercise & Nutrition Sciences, Queensland University of Technology Brisbane Queensland Australia; ^5^ School of Medicine, The University of Queensland Brisbane Queensland Australia; ^6^ Centre for Health Services Research (CHSR), Faculty of Health, Medicine and Behavioural Sciences, The University of Queensland Brisbane Queensland Australia

**Keywords:** burden of illness, child, cost of illness, enteral nutrition, gastric feeding tube, parents

## Abstract

**Background:**

Although pediatric temporary (e.g., nasogastric) feeding tubes are widely used for short‐term nutrition support, family impacts remain poorly defined. Research from long‐term (e.g., gastrostomy) feeding tubes does not generalize because management and burdens differ. This study aimed to explore the financial, time and family burdens of temporary feeding tubes, and their impact on children's quality of life.

**Methods:**

A prospective mixed‐methods longitudinal cohort study was conducted, following families over 4 months. Data were collected using diaries, interviews, Pediatric Quality of Life Inventory (PedsQL^TM^) 4.0 Generic Core Scales, and a caregiver burden questionnaire. Analyses included descriptive statistics for financial and time burdens, a multiple linear regression model fitted to identify factors associated with health‐related quality of life, and qualitative content analysis.

**Results:**

Thirty‐six parents participated. Parents reported spending an average of 3.1 h daily on tube‐related care. Indirect costs (e.g., lost income) averaged USD$1494.15/month (SD = $339.11), and out‐of‐pocket nonmedical costs (e.g., hiring help) represented 44% of monthly family income. Employment disruptions were reported by *n* = 18 (61%) of parents. Key predictors of lower quality of life for children were toddler age group, prior home tube feeding experience, and regional/rural residence. Preterm birth status and involvement of 4–6 medical teams were associated with higher quality of life. Parents reported challenges managing mealtimes, outings, and holidays.

**Conclusion:**

Temporary tube feeding imposes substantial financial burdens, time demands, and disruptions to family life, with impacts on children's quality of life. Supporting successful management for children requiring this intervention requires integrated family‐centered care, including structured support, education, and psychosocial interventions.

## INTRODUCTION

Although feeding tubes deliver much‐needed nutrition support and improvements to growth outcomes, serving as a bridge through feeding difficulties,^1^, ^2^, ^3^, ^4^ they may bring unexpected challenges that impact on family dynamics and resources. These challenges span several domains, including considerable time demands,^5^ substantial emotional and psychosocial impacts on caregivers and children,^6^, ^7^, ^8^, ^9^, ^10^, ^11^ financial burdens,^12^ and difficulties related to the delivery of feeds and consumables.^13^ For families of children with feeding tubes, daily life can involve a complex balancing act of medical caregiving, continuous planning, and navigation of healthcare systems.^14^, ^15^, ^16^

Despite the documented challenges associated with temporary feeding tubes, a critical knowledge gap exists in understanding the specific burdens associated with their use. Understanding the full impact on family functioning is essential for providing comprehensive, family‐centered care. Caregivers describe clinicians as being removed from the realities of home‐based tube feeding care.^17^ Although research to date has explored the impacts of long‐term feeding tubes, such as gastrostomy tubes,^18^, ^19^, ^20^, ^21^, ^22^ fewer studies address challenges specific to temporary feeding tubes (e.g., nasogastric tubes).^23^, ^24^

The burden of caring for children with long‐term feeding tubes (e.g., gastrostomy tubes) manifests across multiple domains of family functioning. Caregivers spend twice as much time caring for a child with a gastrostomy tube compared with a child without a feeding tube.^5^ These time demands can disrupt family mealtimes and strain interpersonal relationships,^17^, ^25^ creating sustained stress for parents.^26^ The psychological impact on parents includes increased risk of anxiety‐depression symptoms,^15^ alongside exhaustion and social isolation.^27^ Notably, these psychosocial challenges stem from tube management rather than the child's underlying medical condition.^26^, ^28^ Financial burden constitutes another considerable burden, with families incurring substantial out‐of‐pocket expenses for supplies and equipment while experiencing reduced work capacity,^7^, ^29^ often exceeding $341 monthly AUD ($223.73 USD).^30^ This multifaceted burden demonstrates how gastrostomy tube management extends beyond standard caregiving into psychological, social, and economic aspects of family life.

Temporary tube feeding can present additional, often unique challenges, including frequent dislodgements, different management (e.g., taping and aspirating), distinct psychological impacts because of their visibility,^31^ different tube duration expectations,^32^ and have been reported to become less acceptable to families over time.^33^ Consequently, children's quality of life may suffer. Temporary feeding tube replacements can impact caregiver‐child relationships^34^, ^35^ and result in physical side effects including gagging, vomiting, and appetite loss,^31^ upsetting the child and further complicating family experiences.^29^, ^36^

Although temporary feeding tubes provide vital short‐term nutrition support, research on their broader family impacts remains limited, despite their frequent use.^37^ They are often promoted primarily as advantageous low‐cost interventions,^38^, ^39^ with the assumption that temporary tubes create proportionally temporary burdens. This may leave families inadequately supported. This study aims to comprehensively evaluate the financial, time, and family burdens experienced by families of children with temporary feeding tubes and assess the impacts on children's quality of life.

## METHODS

### Study design

This study used a prospective convergent parallel mixed‐methods longitudinal design to measure caregiver burden and quality of life for children with temporary feeding tubes over a four‐month time frame, from tube insertion to removal. This research was conducted as part of a larger study examining various aspects and experiences of temporary tube feeding. Data collection included quantitative measures (caregiver burden questionnaire, Pediatric Quality of Life Inventory [PedsQL^TM^] 4.0 Generic Core Scales) and qualitative methods (diaries, interviews) at three key timepoints. Each data set was analyzed separately and then was integrated during interpretation. This allowed for a comprehensive understanding of family experiences. Study design, data collection tools, and interview questions were reviewed by health consumers (parents with lived experience of managing a child with a feeding tube) before commencement to ensure relevance and inclusivity.

#### Health setting and research context

This study was conducted at the Queensland Children's Hospital, a quaternary public children's hospital in Australia. Previous research at this hospital identified that 71 new temporary feeding tubes were placed per month.^40^ The 4‐month timeframe was selected based on typical duration of time a child had a temporary feeding tube managed in this hospital.^40^ In the Australian healthcare system, basic feeding tube consumables (e.g., feeding tubes, syringes, pH strips) and hospital care are provided at no cost to families. Home enteral nutrition is typically government‐subsidized, although families remain responsible for delivery costs and additional items such as specialized tape, feeding equipment, or accessories (e.g., backpacks, poles).

A change in government policy to subsidize home enteral nutrition was implemented in February 2024, towards the end of the data collection period, impacting 12 families. Because this was an unexpected development, collecting information on how this impacted families was not part of the original research design. All participants experienced some period without the subsidy because no families were recruited entirely postimplementation.

#### Ethical approval

Ethical approval was granted by the Children's Health Queensland Hospital and Health Service, Human Research Ethics Committee (Reference number HREC/23/QCHQ/94925) and The University of Queensland Human Research Ethics & Integrity Committee (Reference number: 2022/HE000816).

#### Participants

Parents of children with temporary feeding tubes were recruited using purposeful sampling to ensure diversity in child age, diagnosis, residential location, and previous experience with temporary tube feeding.^41^ For this study, temporary feeding tubes were defined as nasogastric, nasoduodenal or nasojejunal tubes. Eligibility criteria included: parents of children discharged home with a temporary feeding tube; the tube was used for nutrition; parents were aged >18years, living in Australia. With regards to exclusion criteria, this study was focused on children who required temporary tube feeding to improve nutrition for optimal growth. As such, children with diagnosed eating disorders, those requiring tubes solely for medication and children with terminal or uncertain prognoses (e.g., palliative care) were excluded. Non–English‐speaking families and children in foster care without legal guardian consent were excluded. All participants provided written informed consent.

All children were recruited after they had a tube insertion. The majority (*n* = 32) were recruited during hospitalization before discharge home with their feeding tube. Five children were recruited who had already been tube feeding at home before study. For these five children, initial data collection commenced upon their recruitment, ensuring that all participants, regardless of initial tube duration, were followed longitudinally across the defined phases of temporary tube feeding as outlined in Table S1. This approach allowed for a standardized assessment of experiences at comparable points in the temporary tube feeding journey. Data collection phases were tailored to each family's stage of temporary tube feeding (described in Table S1).

### Study procedure

Data were collected at three time points representing key touchpoints in tube feeding care as proposed by Dunitz‐Scheer et al.^42^: tube insertion (during hospital admission), maintenance (once established at home with the feeding tube, typically 1–2 weeks after discharge home), and 4‐months postdischarge or on tube removal, if earlier. Participants maintained a diary for the duration of the study period.

### Data collection

Demographic and clinical data were extracted from medical records to collect information regarding the child's diagnosis, feeding tube details, and home address. Quantitative and qualitative data were collected concurrently at three timepoints (insertion, maintenance, and 4‐month follow‐up), consistent with the convergent parallel mixed‐methods design.

#### Diary data processing and analysis

Parents documented their experiences with their child's feeding tube using an online diary, on a secure online survey platform (Qualtrics®). This diary collected both quantitative and qualitative data using 5‐point Likert scale ratings and open‐ended questions. Topics included daily management challenges, emotional well‐being, time burden, and overall family impact. Additional questions regarding hospital experiences and interactions with clinicians were included in the diary but were not analyzed in this paper because they have a different focus and are planned for a separate review. Regular reminders were sent to ensure consistent diary completion.^43^ Online diaries enabled real‐time, detailed accounts of parent experiences, reducing recall bias and enhancing the richness of the qualitative data collected (see File S1 for diary questions).

#### Semistructured interviews

Semistructured interviews were conducted at each of the three time points using an open‐ended interview schedule (see File S2 for the parent interview guide). This approach allowed exploration of evolving experiences over time while minimizing researcher influence.^44^, ^45^ Although one cost‐related question was included in the initial and final interview, parents independently discussed this topic in other interviews and diary entries. Other interview questions, related to broader experiences, were not analyzed in this study because they have a different focus and are planned for a separate review.

#### Health‐related quality of life assessment

Health‐related quality of life (HRQoL) was measured at each of the three time points, using the Pediatric Quality of Life Inventory (PedsQL^TM^) 4.0 Generic Core Scales, a validated tool for assessing HRQoL in all ages of children, including healthy children and children with acute and chronic conditions.^46^ The scale includes subscales for physical, emotional, social, and daycare/school functioning for children, and cognitive functioning for infants. The PedsQL offers separate scales for children across different ages: 1–12 months, 13–24 months, 2–4 years, 5–7 years, 8–12 years, and 13–18 years. This scale is completed by parents on behalf of the child. Responses were transformed into a 0–100 score, with higher scores indicating better quality of life. All scores were calculated and entered manually into Microsoft Excel, following the guidelines prescribed by Varni^47^ for the scaling and scoring of the results. Scores were checked and entered twice to ensure accuracy of rating. Scoring of a random sample (10%) was checked for accuracy by two separate authors.

#### Caregiver burden questionnaire

A purpose‐built caregiver burden questionnaire was developed because of the absence of validated tools,^48^ to measure time, financial costs, and employment impacts associated with temporary tube feeding (see File S3 for this questionnaire). The questionnaire was informed by a comprehensive literature review.^49^, ^50^, ^51^, ^52^, ^53^ Parents completed this questionnaire at the final time point and were encouraged to report costs related to the temporary feeding tube throughout the study, with a paper copy provided at the beginning of the study for familiarization. Definitions of direct costs, indirect costs, and financial support were based on definitions by Mitterer et al.^49^ These domains included aspects such as additional childcare costs, costs associated with hospital admission, and income loss. All costs in this questionnaire were collected in Australian dollars (AUD). For reporting, values were converted into US dollars using a fixed rate of 0.6561 USD per AUD. The original AUD value results are provided in Table S3.

## DATA ANALYSIS

### Descriptive statistics

Quantitative data, including participant characteristics, financial burden, and time burden, were analyzed using descriptive and inferential statistics. Descriptive statistics were used to summarize participant characteristics and financial data. Continuous variables are presented as means and standard deviations (SD), whereas categorical variables are reported as frequencies and percentages. To ensure analytical stability, categorical variables with low cell counts were collapsed into broader, theoretically relevant groups.

### Statistical analysis

Inferential statistical analyses were used to explore group differences and identify predictors of a child's quality of life (PedsQL scores), and caregivers’ cost‐coping burden and daily tube care time. Covariates for the multivariable linear regression model were selected based on prior literature regarding demographic and clinical factors that may affect a child's HRQoL.^54^, ^55^, ^56^, ^57^, ^58^ As an additional exploratory step, *t* tests and ANOVAs examined bivariate relationships between individual variables and PedsQL scores. These findings informed the final covariates included in the multivariable linear regression model, which aimed to identify independent predictors of PedsQL scores. The final covariates included in the model were as follows: child age (0–12 months, 13–24 months, >25 months), sex (male/female), cultural and linguistic background (Y/N), First Nations background (Y/N), reason for tube insertion (nutrition/growth; unsafe swallow), location (metropolitan, regional/remote), born preterm (Y/N), number of medical teams involved (1; 2–3; 4–6 teams), and prior home tube experience (Y/N). PeDSQL scores were also compared across initial, maintenance and final timepoints using an ANOVA test.

The use of cost‐coping strategies was categorized by the number used (0; 1–3; and 4–8 cost‐coping strategies), and a $\chi^{2}$ test assessed the differences between income groups (<$39,366 per year; $39,366‐65,610 per year; >$65,610 per year). One‐way ANOVA was also used to compare Likert scale ratings between parents who reported spending 1–2 h/day on tube care vs those that were spending >2 h/day on tube care. Statistical significance was set at *P* < 0.05. All analyses were conducted using the statistical software Stata version 17.0 (Stata SE 17; Stata Corp LLC, College Station, TX) and Microsoft Excel Version 16.93.1.

### Qualitative data

Qualitative data from online diaries and semistructured interviews were analyzed using deductive content analysis^59^ with a coding framework based on three predetermined themes: direct costs, indirect costs, and financial support, according to the definitions proposed by Mitterer et al.^49^ Data were segmented into meaning units, coded, and categorized under these themes using NVivo 14 software to ensure systematic organization and analysis. To enhance trustworthiness, initial coding was conducted independently by two researchers, and discrepancies were resolved through discussion. This deductive approach facilitated a convergent integration where qualitative were interpreted alongside quantitative results to provide a comprehensive understanding of family burden and quality of life of children. The interviews conducted for this research had two different objectives: (1) explore the experiences of children with temporary feeding tubes and their families and (2) gather data on the cost burden involved in tube feeding. This article will focus solely on the second objective, with the first objective addressed in a separate research study.

## RESULTS

### Participant characteristics

Thirty‐six parents and thirty seven children were recruited for this study with the additional child accounted for by one set of twins described in Tables 1 and 2. Demographic data were collected from 34 parents after two were lost to follow‐up immediately after recruitment. Most identified as mothers (*n* = 31; 91%). Of the 36 parents enrolled, 28 (77%) completed all phases of the study, although one parent did not complete any diary entries during their participation. In total, nine parents in total were lost to follow‐up, two withdrew because of time commitments, and seven could not be contacted within the study timeframe. Table 1 summarizes participant retention and data collection across study phases, noting varying sample sizes for analysis because of attrition at later timepoints.

**Table 1:** Parent and family characteristics (*n* = 34).

Category	*n* (%)
Age of primary caregiver at child's tube insertion	
20–30 years	13 (38%)
31–45 years	21 (62%)
Working status of primary caregiver	
Currently employed	8 (24%)
On leave	2 (6%)
Maternity leave	8 (24%)
Not working	15 (44%)
Student	1 (3%)
Highest level of education of primary caregiver	
Year 12 and below	11 (32%)
Professional qualification (e.g., diploma)	8 (24%)
University qualification	15 (44%)
Household	
Siblings at home	22 (65%)
Single parent family	2 (6%)
Number of people in household (mean ± SD)	4.3 ± 1.3
Background	
First Nations	4 (12%)
Culturally and linguistically diverse	6 (18%)
Geographical location	
Metropolitan	26 (70%)
Regional	10 (27%)
Remote	1 (3%)

**Table 2:** Characteristics of children (*n* = 37).

Category	*n* (%)
Tube type	
Nasogastric	36 (97%)
Transpyloric	1 (3%)
Feeding tube use (at home) before study participation	
Yes	5 (14%)
No	32 (86%)
Tube feeding method	
Bolus	22 (59%)
Continuous	8 (22%)
Combination of continuous and bolus	7 (19%)
Tube use at study completion (*n* = 28)	
Tube remained in place	15 (54%)
Tube was removed	13 (46%)
Primary reason for tube insertion	
Nutrition/growth	28 (76%)
Unsafe swallow/nil by mouth	9 (24%)
Primary diagnosis categories	
Circulatory/cardiac conditions	8 (22%)
Oncological conditions	8 (22%)
Other medical conditions	21 (56%)

Children (19 males [51%]; 18 females [49%]) had an average age of 30.7 months (SD = 52.4) at tube insertion. Most had nasogastric tubes (97%; *n* = 36), primarily for growth and nutrition purposes (76%; *n* = 28), including those placed because of an unsafe swallow (24%; *n* = 9). Circulatory disorders and neoplasms were the most common diagnoses. Children who already had feeding tubes at home before recruitment (*n* = 5) had longer tube durations, averaging 5.6 months (SD = 3.6, range 1–10 months), compared with children who had not yet been home with their tubes (mean = 1.6 months; SD = 2.6).

Notably, 41% of parents had a university‐level education (*n* = 14), and 47% of parents reported that the primary caregiver was not currently working (*n* = 16). Families met diverse purposeful sampling criteria, including those with First Nations background (12%, *n* = 4), those with siblings (65%, *n* = 22), families from both metropolitan and regional/rural areas (30%, *n* = 11), and households with single parents (6%, *n* = 2). Parent participants completed 81 interviews and 223 diary entries documenting their experiences over the study period. Parent participants are referred to using the format of “*P*[number]” (e.g., P21).

#### Financial burden

Amounts are described in US dollars, converted using a fixed rate of 0.6561 USD per AUD. Original Australian dollar result values are provided in Table S3. The financial impact of temporary feeding tubes presented a significant challenge for families, encompassing both direct and indirect costs, described in Tables 3 and 4. Representative parent quotes illustrating these financial burdens are provided in Table S2. Twenty‐eight parent participants completed the caregiver burden questionnaire at the study completion. Participants completed only questionnaire items relevant to their circumstances, resulting in varying response rates for individual cost categories (e.g., not all families hired additional help or purchased extra clothing).

**Table 3:** Direct costs related to temporary tube feeding (*n* = 28).

Category	*n*	Mean (±SD)	Total monthly cost^a^ (Mean ± SD)
**Annual family income**			
$0–39,366	7		
$39,367–65,610	6		
$65,610 and above	12		
**Out‐of‐pocket direct medical costs**			**$589.53 ± 218.11**
Total medical costs related to feeding tube	25	$207.54 (±180.69)	
Treatment costs (e.g., GP fees)	22	$262.69 (±186.94)	
Equipment for the tube	23	$84.35 (±169.94)	
**Out‐of‐pocket direct non‐medical costs**			**$1494.15 ± 657.59**
Hiring help (e.g., carers)	4	$147.79 (±217.99)	
Communication (e.g., increased phone use)	13	$58.16 (±177.01)	
Hygiene (e.g., extra laundry)	16	$90.40 (±169.22)	
Buying extra food (e.g., for hospital visits)	20	$180.72 (±132.52)	
Buying extra clothes	14	$84.50 (±386.95)	
Hiring a cleaner	1	$32.80 (±0)	
**Inpatient costs**			**$544.92 ± 218.11**
Extra essential products (e.g., pillows)	10	$117.89 (±169.62)	
Accommodation for inpatient stay	4	$229.96 (±15.33)	
Extra childcare during inpatient stay	4	$197.07 (±136.27)	
**Outpatient costs**			**$354.85 ± 331.78**
Extra gifts, treats, and toys	10	$118.29 (±160.68)	
Essential outpatient products	5	$72.37 (±193.59)	
Relocation (to be closer to hospital)	2	$164.19 (±216.29)	

*Note*: Reference used: Board of Governors of the Federal Reserve System. **Foreign Exchange Rates (H.10): U.S. dollars per Australian dollar.** Washington, DC: Board of Governors of the Federal Reserve System. Accessed 13 October 2025: https://www.federalreserve.gov/RELEASES/H10/hist/dat00_al.htm.

Abbreviation: GP, general practitioner.

^a^ Dollar amounts are USD. Currency conversion used a fixed rate of 0.6561 US dollars per Australian dollar, reflecting the 2023‐2024 study period.

**Table 4:** Indirect costs, time burdens and employment impacts (*n* = 28) in USD.

Category	*N*	Value^a^
**Indirect costs (income loss)**	*n*	
Primary caregiver	26	$380.80 (±242.20)
Partner	8	$459.60 (±237.34)
Total monthly cost		**$840.40 (±339.11)**
**Financial support received**	***N*** **(%)**	
Received home enteral nutrition at reduced cost	5	‐
Had NDIS funding	2	‐
Received non‐government support	7	‐
Received government support	19	‐
**Employment impact on primary caregiver**	***N*** **(%)**	
Unchanged	8	‐
Stopped working	10	‐
Took leave/reduced work hours	6	‐
Turned down a promotion/pay raise	2	‐
Decreased salary	10	‐
**Employment impact on partner**	***N*** **(%)**	
Unchanged	13	‐
Stopped working	3	‐
Took leave/reduced work hours	7	‐
Turned down a promotion/pay raise	3	‐
Decreased salary	8	‐
**Time commitments**	n	
Tube feeding tasks	27	21.7
Attending tube‐related appointments	26	1.8
Travel to tube‐related appointments	27	2.3
Communicating with clinicians	27	1.3

*Note*: Reference used: Board of Governors of the Federal Reserve System. **Foreign Exchange Rates (H.10): U.S. dollars per Australian dollar.** Washington, DC: Board of Governors of the Federal Reserve System. Accessed 13 October 2025: https://www.federalreserve.gov/RELEASES/H10/hist/dat00_al.htm.

Abbreviation: NDIS, National Disability Insurance Scheme.

^a^ Dollar amounts are USD. Currency conversion used a fixed rate of 0.6561 US dollars per Australian dollar, reflecting the 2023‐2024 study period.

##### Direct out‐of‐pocket medical costs

Costs incurred included tube supplies, equipment and feeds, averaging $589.53 (SD: $218.11) monthly for families. Of the 24 parents who provided out‐of‐pocket cost data in the questionnaire, those who received the government subsidy (*n* = 11) reported mean costs of USD $215.20 (SD: $172.55) compared with USD $201.42 (SD: $193.55) for those without the subsidy (*n* = 13); no statistical test was conducted because of the small sample size. In the interviews many parents reported unexpected and out‐of‐pocket costs, including supplies such as feeding poles, which were difficult to source and cost “*hundreds of dollars*” [P20]. Even routine care required improvisation and extra costs, with a parent reporting they “*had to go out and buy a pliers*” [P13] to open the feeding tube cap. These extra costs accumulated for parents over time, with regular purchases of bottles, syringes, tubes, and tape, compounded by inadequate hospital provision requiring them to buy additional supplies. In addition, unclear processes regarding hospital supplies caused further stress and confusion, leaving parents unable to determine which supplies were provided free and which would require payment.

##### Direct out‐of‐pocket nonmedical costs

Direct non‐medical costs averaged $1,494.15 (SD: $657.59) per month, and the total cost of travel to and from the hospital was $303.75 (SD: $172.14). Additional to equipment in the interviews, several families reported paying private clinicians to help manage their child's feeding tube. Location mattered and added with families having to pay premium costs to have tube feeds shipped directly when they went on trips. Although some families did not report experiencing financial strain, through fortunate circumstances such as having healthcare backgrounds or personal connections to clinicians that helped, most acknowledged the considerable impact potential: “*[the cost] wasn't an issue for us, but I can see that it might be an issue for other people*” [P17].

##### Indirect costs

A marked impact was observed in disruption to parents’ livelihoods through reduced working hours or leaving jobs entirely, with lost income averaging $840.40 (SD: $339.11) per month. The average annual (net) family income was $41,006.25, although seven (28%) families had an annual income of <$39,366, and almost half (*n* = 12, 48%) earned over $65,610. Considering the average reported monthly family income was $3416.97, based on these figures, families experienced a 25% income loss and spent 44% of their income on out‐of‐pocket tube‐related costs per month. Employment disruption was widespread, affecting 61% (*n* = 18) of primary caregivers and 51% (*n* = 13) of partners, with 38% (*n* = 10) of primary caregivers stopping work entirely. Findings also documented notable income losses among friends and relatives who provided informal caregiving support. Nine parents reported that these informal caregivers lost an average of $594.46 (SD: $374.93) per month in income. Although 76% (*n* = 19) of families received various forms of government support, only one parent reported having income protection during leave from work.

The requirements for temporary tube feeding care created difficult choices for parents. The specialized nature of tube feeding tasks limited which family members could provide care, directly impacting both parents’ ability to work. Financial pressures further complicated these decisions, with some parents unable to access carer's payments and so needed to continue working despite their child's care needs. The cumulative medical caregiving burden on parents felt “*like a full‐time job, and it's not cheap*” [P21].

##### Financial support

Financial assistance varied considerably and seemed to be based largely on parents’ system literacy and advocacy skills. Some secured support from National Disability Insurance Scheme (NDIS) funding, a government‐funded program providing support to those with disabilities, whereas others discovered benefits only through persistence. However, even when financial assistance was available, the cumulative impact remained substantial for many families.

##### Cost‐coping strategies

Families employed various strategies to manage the financial burden of tube feeding. Most parents (60%, *n* = 21) reported using cost‐coping strategies, and of these, 12 (34.3%) used 1–3 strategies, and 9 (25.7%) used 4–8 strategies. The most common cost‐coping strategies included using savings (*n* = 17), reducing leisure activities (e.g., eating out) (*n* = 14), reducing spending on basic needs like food (*n* = 11) and borrowing money/using a credit card (*n* = 7). The number of cost‐coping strategies used was not significantly different across different income levels (χ^2^ = 4.471; *P* = 0.346), suggesting families across all income brackets faced similar challenges in managing expenses.

#### Family burden

Family burden encompassed time demands and day‐to‐day impacts on family routines. Results are presented in two subdomains: time burden and daily life management.

##### Time burden

**Figure 1:**
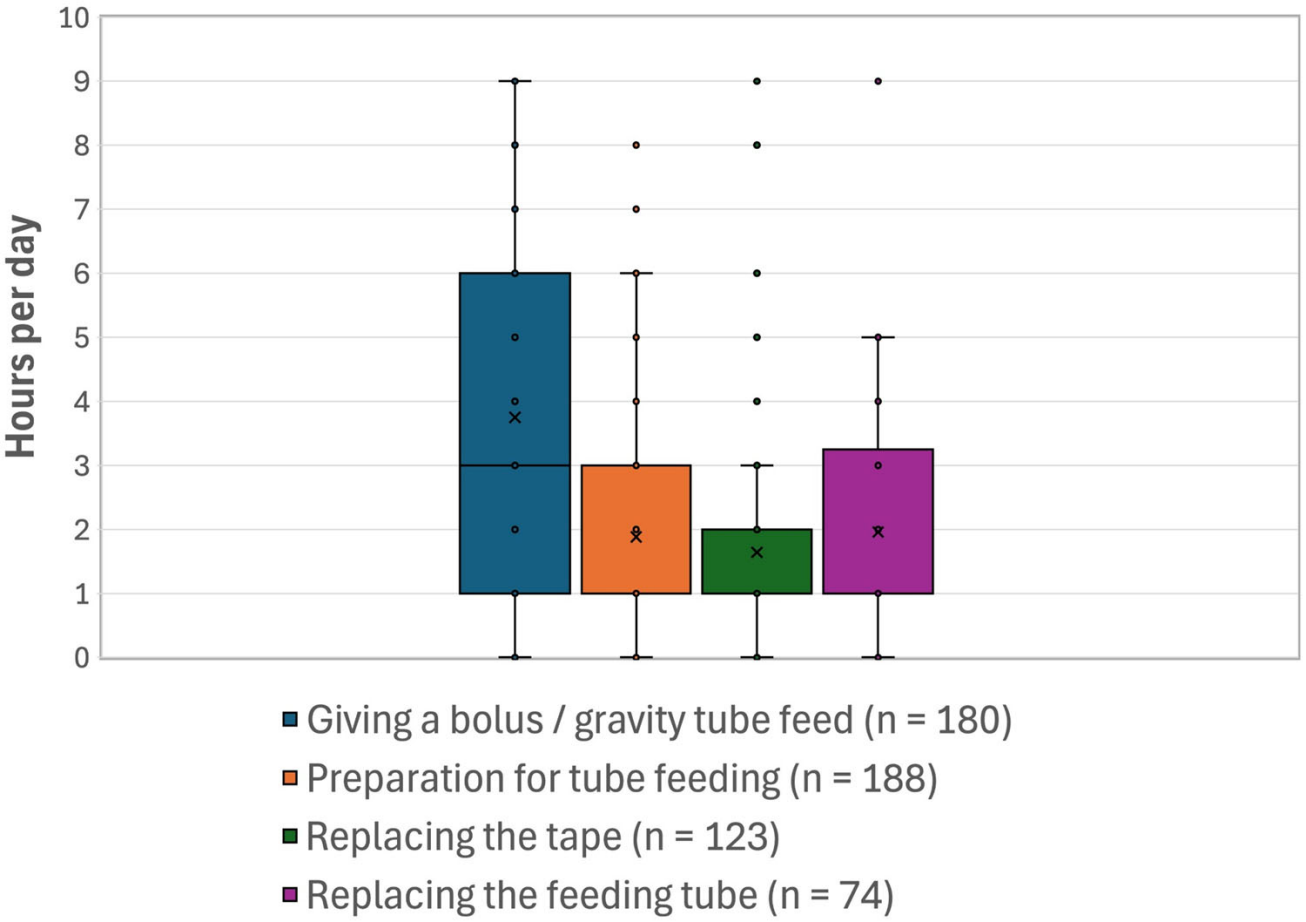
Daily time commitment of parents to tube feeding tasks. The “*n*” value beside each task represents the number of distinct parents who reported data for that specific task's duration over the course of their child's participation in the study. Boxes represent the interquartile range (25th to 75th percentile), with the horizontal line indicating the median. The “x” marks the mean. Individual dots represent outliers.

Parents reported spending substantial amounts of time on tasks related to their child's feeding tube. In the burden questionnaire (Table 4), parents provided retrospective estimates of the time spent on tube‐related tasks. The greatest time commitment was for daily tube care (mean = 3.1 h/day), with additional weekly time spent attending appointments, travelling, and communicating with clinicians. Parent diary data (Figure 1) further highlighted the considerable daily effort required for tube feeding care, with giving tube feeds, preparation, tape changes, and tube replacements taking multiple hours each day. One parent shared, “*It took three attempts to get his new tube in this week, it wouldn't work and he was screaming and getting really worked up, it took a long time*” [P08].

##### Daily life management

**Figure 2:**
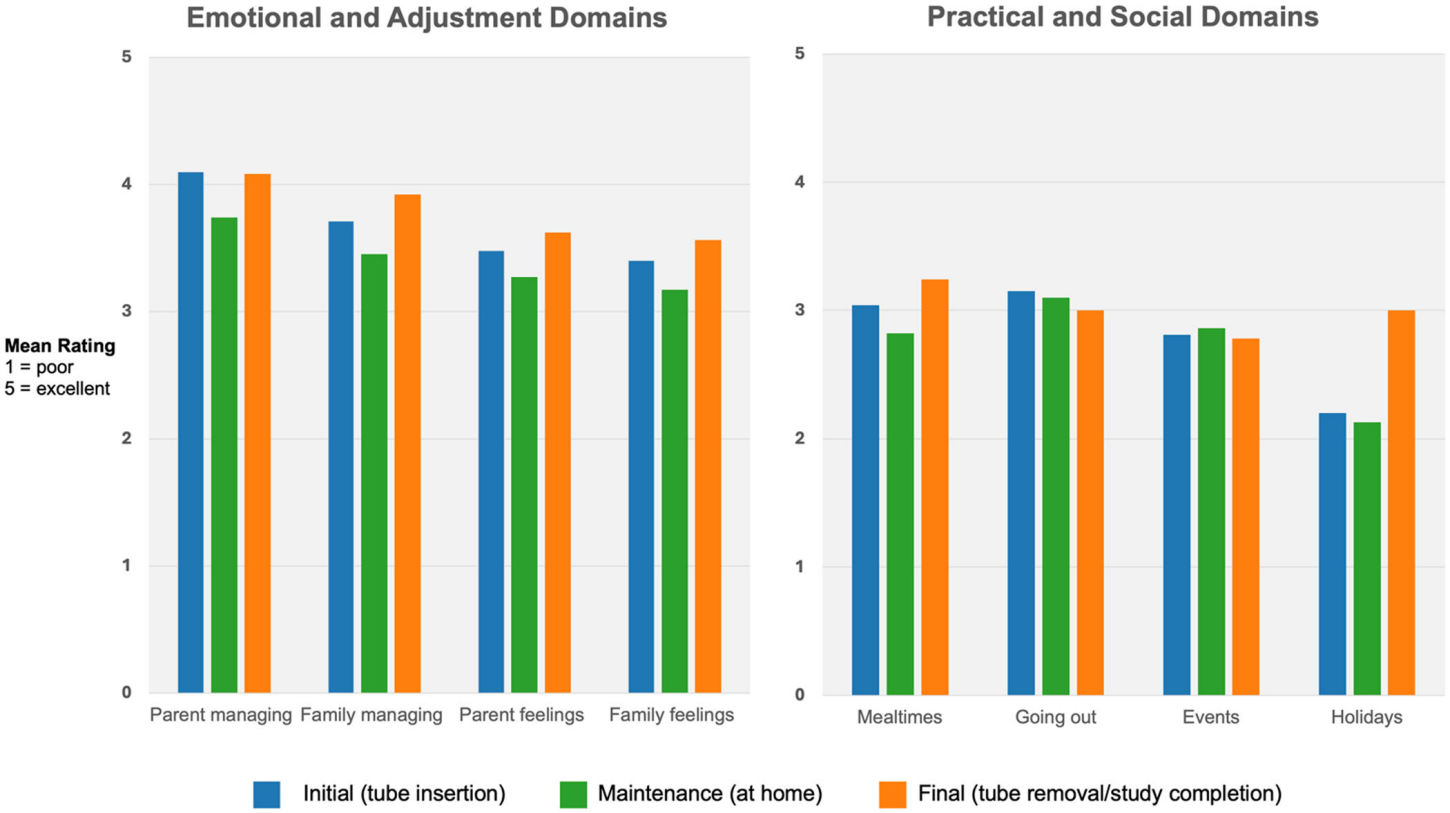
Parental perceptions of daily life and adjustment across the phases of temporary tube feeding. Parental perceptions of daily life and adjustment across tube feeding phases. Mean ratings (1 = not well at all; 5 = extremely well) are shown by phase (initial, maintenance, and final) across emotional/adjustment (left) and practical/social (right) domains.

Quantitative diary data captured how families managed their daily life across emotional, practical and social domains (Figure 2). Across the study period, 32 parents completed at least one diary entry. Parents showed high completion rates for the Likert scale questions, answering an average of 5.7 out of 6 questions per diary entry. To examine trends over time, diary entries were grouped into three phases: initial (entries submitted before the first maintenance interview, or the first entry if no interview occurred); maintenance (entries after the maintenance interview, or those between the first and last entries when no interview was conducted); and final (each parent participant's last diary entry). Single‐entry participants were categorized as maintenance for consistency.

Parents reported higher mean scores for questions regarding tube management, indicating more positive ratings. Lower scores were provided with regards to how the feeding tube impacted other areas of their lives, such as going out in public. Differences between ratings across items were not statistically significant, with most scores averaging near the midpoint (approximately 3/5), indicating parents viewed these aspects as neither particularly easy nor difficult. No significant differences between the timepoints were observed. One‐way ANOVA was conducted to explore whether parents who spent more time managing their child's feeding tube per day (1–2 h vs >2 h) reported greater impact on daily life activities, such as mealtimes, going out in public, attending events, and going on holidays. Parents in the high time‐burden group (>2 h/day) generally reported a greater impact on these activities than those in the low time‐burden group (1–2 h/day), but these differences were not statistically significant. Unsurprisingly, parents who devoted more time to tube management reported experiencing greater disruption across multiple domains of daily life because the disproportionate time commitment to feeding care inevitably reduced time available for other family activities, mealtimes, and outings.

#### Child‐related burden

Child PedsQL quality‐of‐life outcomes, univariate comparisons (*t* tests/ANOVAs), and the adjusted regression model are summarized in Tables 5 and 6, respectively.

**Table 5:** Child quality of life PedsQL Scores (*t* tests and ANOVAs).

Variable	No. of observations	Mean ± SD)	*P* value
Sex			
Male	36	58.39** ± **2.72	0.3426
Female	49	60.02** ± **2.79	
Remoteness location			
Metropolitan	55	61.75** ± **2.37	0.0485
Regional/remote	30	54.86** ± **3.42	
Background			
First Nations	12	59.24** ± **2.02	0.3059
Non‐First Nations	72	62.12** ± **6.80	0.3059
Cultural and Linguistically Diverse	14	59.16** ± **2.25	
Non‐ Cultural and Linguistically Diverse	71	59.16** ± **3.65	0.9695
Born preterm			
Yes	17	61.01** ± **4.25	0.3369
No/Unsure	68	58.9** ± **2.23	
Age of child at tube insertion			
0–12 months	56	64.97** ± **2.13	0.0000*
>13 months	29	48.44** ± **3.23	
Reason for tube insertion			
Nutrition/Growth	67	59.65** ± **17.19	0.3804
Unsafe swallow	18	58.16** ± **5.20	
Child had been home with the tube before study commencement			
Yes	23	45.02** ± **2.28	0.0000*
No	60	65.34** ± **2.20	
Number of medical teams involved in a child's care			
1 team	14	58.33** ± **16.68	0.622
2–3 teams	16	59.14** ± **19.76	
4–6 teams	7	61.58** ± **18.7	
Child age			
0–12 months	23	67.98** ± **15.65	0.030*
13–24 months	4	52.50** ± **9.26	
>25 months	10	45.63** ± **18.84	
Across time			
Initial	32	52.98** ± **17.41	0.031*
Maintenance	25	61.14** ± **16.23	
Final	28	64.97** ± **19.07	

*Note*: *Significant results of *P* < 0.05. Observations represent repeated measures from the same children across multiple timepoints (initial, maintenance, final phases of temporary tube feeding). PedsQL scores are on a 0–100 scale, with higher scores indicating a higher quality of life. ‘Number of medical teams involved in a child's care’ refers to the number of subspecialty teams actively engaged in a child's care. These included: General Pediatrics, Neonatology, Intensive Care, Surgery, Oncology, Haematology, Metabolic Medicine, Nephrology, Neurology, Infectious Diseases, Ophthalmology, Oral and Maxillofacial Surgery, Orthopaedics, Palliative Care, Rehabilitation, Respiratory, and Rheumatology.

**Table 6:** Child quality of life PedsQL scores (regression analysis).

Variable	Adjusted regression model, Β (95% CI), *P* value
**Child age**	
0–12 months (reference)	
13–24 months	−15.187, (−28.498 to −1.875), 0.025*
>25 months	−12.885 (−31.205 to 5.433), 0.168
**Age of child at tube insertion**	
0–12 months (reference)	
13 months and above	8.071, (−8.049 to 24.192), 0.326
**Sex**	
Male (reference)	
Female	−4.898, (−15.194 to 5.397), 0.351
**First Nations background**	
Yes (reference)	
No	−0.351, (−16.047 to 15.345), 0.965
**Culturally and linguistically diverse background**	
Yes (reference)	
No	−2.865, (−13.805 to 8.073), 0.608
**Remoteness location**	
Metropolitan (reference)	
Regional/remote	−18.866, (−30.285 to −7.447), 0.001*
**Born preterm**	
No (reference)	
Yes	20.966, (4.141 to 37.792), 0.015*
**Reason for tube insertion**	
Nutrition/Growth (reference)	
Unsafe swallow	0.970 (−12.282 to 14.223), 0.886
**Child had been home with the tube before study commencement**	
No (reference)	
Yes	−20.482, (−30.139 to −10.825), 0.000*
**Number of medical teams involved in a child's care**	
1 team (reference)	
2–3 teams	−12.020 (−25.716 to 1.675), 0.085
4–6 teams	69.682, (60.102 to 79.262), 0.000*

*Note*: *Significant results of *P* < 0.05; negative values for B indicated a lower PedQLs score. PedsQL scores are on a 0–100 scale, with lower scores indicating a lower quality of life. ‘Number of medical teams involved in a child's care’ refers to the number of subspecialty teams actively engaged in a child's care. These included: General Pediatrics, Neonatology, Intensive Care, Surgery, Oncology, Haematology, Metabolic Medicine, Nephrology, Neurology, Infectious Diseases, Ophthalmology, Oral and Maxillofacial Surgery, Orthopaedics, Palliative Care, Rehabilitation, Respiratory, and Rheumatology.

##### Geographic factors

An independent samples *t* test showed that children living in regional and remote areas reported markedly lower quality of life scores compared with those in metropolitan areas (mean difference = 6.89; *P* < 0.05). This geographic disparity remained significant even after controlling for other variables in regression analysis (*β* = −18.87; 95% CI, −30.29 to −7.45; *P* = 0.001) such as reason for tube feeding, age, preterm status, and cultural background.

##### Age‐related differences

A one‐way ANOVA indicated that younger children had significantly better quality of life, with infants (0–12 months) demonstrating higher scores (mean = 67.98; SD: 15.65) than both toddlers (13–24 months; mean = 52.50; SD: 9.26) and older children (>25 months; mean = 45.63; SD = 18.84) (*P* = 0.030). Further regression analysis adjusting for key variables demonstrated that children aged 13–24 months experienced significantly reduced quality of life compared with infants (*β* = −15.19; 95% CI, −28.50 to −1.88; *P* = 0.025).

##### Prior experience with feeding tubes

A *t* test demonstrated that children who had been home with a feeding tube befoee study commencement and therefore had a longer duration of tube feeding had substantially lower quality of life scores (mean = 45.02; SD: 2.28) compared with those that had not been home with a tube and had a shorter tube duration (mean = 65.34; SD: 2.20; mean difference = 20.32; *P* < 0.001). Regression analysis further supported this, showing a strong negative association, in that longer tube feeding duration was associated with lower quality of life scores (*β* = −20.48; 95% CI, −30.14 to −10.83; *P* < 0.001). Notably, children with prior experience of tube feeding at home showed comparable medical complexity to those that did not, with only a small, nonsignificant difference in the number of medical teams involved (3.0 ± 2.3 vs 2.4 ± 1.5 teams respectively; *P* > 0.5).

##### Protective factors

Two factors were associated with higher reported quality of life. Multivariate regression analysis found preterm birth was associated with significantly better quality of life scores (*β* = 20.97; 95% CI, 4.14–37.79; *P* = 0.015). Children with involvement from 4–6 medical teams also had significantly higher quality of life scores compared with those with only one team involved (*β* = 69.68; 95% CI, 60.10–79.26; *P* < 0.001).

##### Longitudinal improvement

A one‐way ANOVA showed that quality of life scores improved significantly over the study period (*P* = 0.031), from the initial time point (mean = 52.98; SD: 17.41) to maintenance (mean = 61.14; SD: 16.23) and final time points (mean = 64.97; SD: 19.07). Post hoc pairwise comparisons indicated that the greatest improvement occurred between the initial and final timepoints, suggesting a gradual yet meaningful increase in reported quality of life over time. However, even at study completion, scores remained considerably well below age‐matched normative data of 82.5,^60^ highlighting the persistent impacts of temporary feeding tubes on children's well‐being.

## DISCUSSION

Temporary feeding tubes provide life‐sustaining nutrition for children navigating feeding challenges with their use and benefits well established in clinical practice. This study revealed that temporary tube feeding can be associated with substantial financial strain, significant time demands, and reduced quality of life for children. Geographic remoteness, child age, and prolonged temporary feeding tube duration magnified impacts on children's quality of life, whereas premature birth and multiteam involvement may have offered some protection. These findings challenge general assumptions of temporary feeding tubes as low‐cost, minimally invasive interventions and underscore the need for structured family support systems to underpin their use.

### The interconnected nature of burden

**Figure 3:**
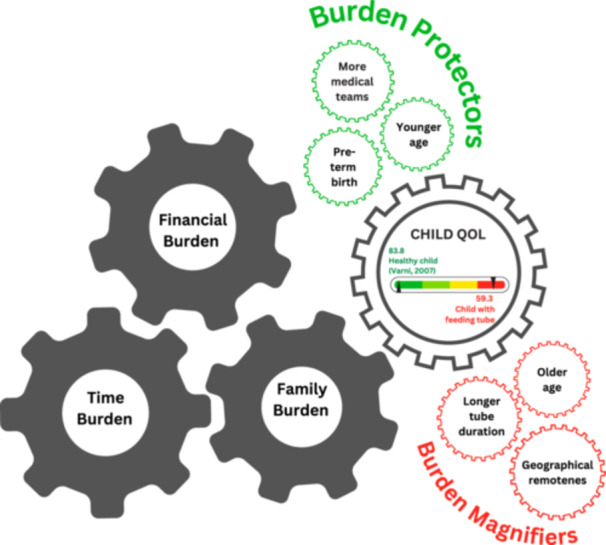
Interconnected burdens and influencing factors related to temporary tube feeding. This figure illustrates the interconnected burdens of financial, time, family, and child quality of life, alongside contextual factors that may act as protectors or magnifiers of a child's quality of life. QOL = quality of life.

Temporary feeding tubes create a complex, self‐reinforcing network of burdens that challenges traditional assumptions that these devices are low‐impact interventions, as shown in Figure 3. These findings extend previous research on the multifaceted nature of caregiving burden,^61^ by revealing the unique temporal dimension of temporary tube feeding. Although financial burden is well‐documented in families of technology‐dependent children,^62^, ^63^ this study found that 60% of families adopted cost‐coping strategies, regardless of income. Critically, the financial burden was closely linked with other forms of burden. Employment instability, reported by many parents, was compounded by the time demands of tube feeding tasks averaging 3.1 h per day. These competing demands may have restricted work capacity and contributed to indirect income loss; a burden families seldom reported explicitly despite its substantial impact. Beyond financial strain, time burden disrupted the rhythm of family life. Parent diary data revealed difficulties with planning outings, managing mealtimes and going on holidays. These ongoing disruptions threaten parental mental health because previous research demonstrates that time scarcity and unpredictability can intensify caregiver anxiety, social withdrawal, and loss of normality.^7^, ^26^, ^28^, ^64^

Additionally, these interconnected burdens were not contained within the nuclear family. Informal caregiving by relatives and friends imposed considerable personal cost, an invisible cost hidden in traditional care planning. Sociodemographic context further shaped burden severity. In this study, over half the parents had lower education levels and incomes, factors previously associated with heightened caregiving strain in parents caring for a child with a gastrostomy tube.^25^ Unlike long‐term feeding tubes, temporary tubes are associated with ongoing uncertainty through frequent tape replacement, unplanned tube reinsertions, and unclear duration. This unpredictability may have limited families’ ability to adapt and stabilize, perpetuating their stress over time. Together, these findings formed a feedback loop that intensified overall burden and risk of financial toxicity.^65^ These findings highlight the need for coordinated models of care that proactively address the interlinked financial, temporal, and psychosocial burdens experienced by children and their families. Embedding burden assessments into discharge planning, improving access to family support, and implementing routine financial screening may help disrupt this cycle of burden and improve family well‐being.

### Systemic gaps in support

Inconsistent financial support structures further sustains this network of interconnected burdens experienced by families, aligning with previous research findings.^66^ In this study, many parents described that access to financial support was reliant on their own advocacy, prior experience, or their clinical background, highlighting a lack of structured hospital support. Where this research was undertaken, dietitians were responsible for referring to social work to enable reduced‐cost home enteral nutrition scripts, yet only a minority of families received them in this study. Some parents had to explicitly request support, indicating that eligibility alone was insufficient. These findings reflect broader systemic gaps. An audit conducted at the same hospital indicated that only a small proportion of children with temporary feeding tubes were referred to social work,^67^ suggesting that families’ access to financial assistance may be variable. This gap is further compounded by the absence of routine financial needs assessments,^66^ despite evidence that parents are receptive to this type of hospital‐based screening.^68^ These findings reinforce ongoing calls for national policy guidance and clinical standards for pediatric tube feeding.^30^, ^69^ Clearer communication about available subsidies and structured financial assistance programs could alleviate the financial strain.^15^ Embedding routine financial eligibility assessments into discharge planning would ensure that support reaches all eligible families, not just those most informed or assertive.

### Quality of life of children: impacts and influencing factors

Children with temporary feeding tubes experienced dramatically lower quality of life scores than their healthy peers and children with other medical conditions, including oncology, poststroke, and end‐stage renal disease diagnoses.^60^, ^70^, ^71^, ^72^ This finding challenges fundamental assumptions that temporary feeding tubes represent a minor medical intervention. Despite documented evidence of these tubes causing significant pain, gagging, vomiting, and anxiety,^29^, ^31^, ^73^, ^74^, ^75^ children with temporary tubes are rarely recognized as a high‐burden population requiring specialized support. The longitudinal quality of life data revealed concerning patterns that persisted beyond initial tube placement. Quality of life was lowest immediately after tube insertion, improved gradually over time, but never returned to normal levels throughout the study period.^60^ Children have consistently described having temporary feeding tubes as frightening, embarrassing, and socially isolating,^76^, ^77^, ^78^ yet their perspectives remain largely absent from care planning decisions.

Specific factors either amplified or protected against quality‐of‐life impacts. Children in regional and rural areas experienced significantly lower well‐being scores than their metropolitan peers, likely reflecting reduced healthcare infrastructure, fewer specialists, and higher travel‐related costs.^79^, ^80^ Conversely, children born preterm demonstrated higher quality of life scores, possibly because of parents’ adjusted developmental expectations^81^, ^82^ and comprehensive neonatal support systems.^83^, ^84^, ^85^ Involvement of multiple medical teams (4–6) unexpectedly correlated with better outcomes, suggesting that coordinated multidisciplinary care may buffer against quality of life impacts through consistent follow‐up care and comprehensive support.^86^, ^87^ These findings demand immediate healthcare system responses. The persistent nature of quality of life impacts indicates that support must extend well beyond initial hospital discharge. Healthcare systems must routinely screen for child well‐being, integrate structured peer support and mental health services, and prioritize children's voices in feeding tube decisions to prevent lasting developmental impacts.

### Limitations

This study's small sample size may limit generalisability, and findings from one hospital system might not capture variations in funding or clinical protocols across different settings. However, regional and rural families who received care from other hospitals faced similar challenges, suggesting wider applicability beyond the recruiting hospital. Additionally, some reported burdens may be attributable to the child's overall health needs rather than solely the temporary feeding tube. Efforts were made to minimize this influence through focused questions about tube‐related activities specifically. During the study, the state government introduced free home enteral nutrition, affecting a third of participating families. Only 24 parents (11 that received the subsidy and 13 that did not) provided out‐of‐pocket cost data in the burden questionnaire, so statistical analysis was under‐powered, and the observed mean difference (13.12) was modest. Although preliminary, this suggests that subsidizing nutrition alone fails to address the broader multifaceted financial burdens these families experience. Finally, quality‐of‐life scales were completed by parents on behalf of their children, presenting potential bias. Although the PedsQL is a valid proxy‐report scale with proven reliability, self‐report has been considered the gold standard.^88^

### Future directions

Future research could extend the follow‐up period, capture broader demographic diversity, and explore validated burden‐of‐care tools. Larger, multicenter studies could examine how targeted interventions, such as telehealth or comprehensive discharge education affect caregiver burden and children's quality of life. Research must specifically examine how targeted interventions across multiple domains, including financial, emotional, and practical domains, could effectively lighten the substantial burden these families manage while preserving the clinical benefits of temporary tube feeding for children.

## CONCLUSION

Temporary tube feeding provides essential and often life‐saving nutrition support to children. However, these benefits must be balanced with the substantial financial, and logistical strain they place on families, as well as the potential impacts on a child's quality of life. This study establishes temporary feeding tubes as distinct from long‐term feeding tubes with unique burden patterns requiring tailored support. Alleviating these burdens will depend on integrating structured education, psychosocial support, and financial assistance within a family‐centered model of care. Recognizing the clinical and psychosocial impacts of temporary feeding tubes is essential to ensure care is both effective and sustainable for families.

## AUTHOR CONTRIBUTIONS

Claire Reilly, Rebecca Packer, Jeanne Marshall, and Nikhil Thapar contributed to the conception and design of the research. Claire Reilly, Jeanne Marshall, Jasmine Foley, and Syed Afroz Keramat contributed to the acquisition and analysis of the data. Claire Reilly, Jasmine Foley, and Jeanne Marshall drafted the manuscript. Claire Reilly, Jasmine Foley, Rebecca Packer, Jeanne Marshall, Syed Afroz Keramat, and Nikhil Thapar critically revised the manuscript for important intellectual content. Claire Reilly, Jasmine Foley, Rebecca Packer, Jeanne Marshall, Syed Afroz Keramat, and Nikhil Thapar agree to be fully accountable for ensuring the integrity and accuracy of the work and have read and approved the final manuscript.

## CONFLICT OF INTEREST STATEMENT

The authors declare no conflicts of interest.

## ETHICS STATEMENT

Ethics approval was obtained from the Children's Health Queensland Hospital and Health Service, Human Research Ethics Committee (Reference number HREC/23/QCHQ/94925) and The University of Queensland Human Research Ethics & Integrity Committee (Reference number: 2022/HE000816).

## Supporting information

Table S1 **‐** Participant retention and data collected by phase of temporary tube feeding.
Table S2 ‐ Representative parent quotes illustrating financial burdens. Table S3 ‐ Reported results in Australian dollars (AUD).
File S1 ‐ Diary template.
File S2 ‐ Parent interview guide.
File S3 – Caregiver burden questionnaire.
