# Supplementary material for: More than meets the eye: The hidden burden of temporary feeding tubes on children and their families
Source: Nutr Clin Pract. 2025 Oct 26;41(2):662–79. doi: 10.1002/ncp.70048 (PMC12982654; doi:10.1002/ncp.70048)
Supplement: Supplementary file 1 — Table S1 ‐ Participant retention and data collected by phase of temporary tube feeding. Table S2 ‐ Representative parent quotes illustrating financial burdens. Table S3 ‐ Reported results in Australian dollars (AUD). File S1 ‐ Diary template. File S2 ‐ Parent interview guide. File S3 – Caregiver burden questionnaire. [file NCP-41-662-s001.pdf]

File S1. Diary Template

Section 1: Managing the Feeding Tube

Who is completing this diary?
Child

Mother

Father

Other family member/caregiver (please specify)

Please rate the following on a scale of 1 (terrible) to 5 (great):

How are you managing the feeding tube? (e.g., using the tube, replacing the tape)

How is your family managing with the feeding tube?

How are you feeling about the feeding tube?

How is your family feeling about the feeding tube?

Section 2: Daily Routines

In the past week, how well have the following activities been going? (1 = not well at all, 5 = extremely well)
Going to school/daycare

Meal times

Going out in public (e.g., shopping, park)

Attending events (e.g., birthdays, family dinners)

Going on holidays

Section 3: Time Spent on Tube Feeding

On average, how many hours per day does each of the following take? (Select 0–9 hours)
Giving a tube feed

Preparing the feeding tube (e.g., syringe setup, pH strip, feeding pump)

Replacing the tape to secure the feeding tube

Replacing the feeding tube

Please share if anything was different at home with the feeding tube in the last week (e.g., tube needed replacing multiple times):

Section 4: Healthcare Interactions

Have you had any allied health reviews about the feeding tube? (Tick all that apply)

Dietitian

Speech Pathologist

Occupational Therapist

Feeding Therapy Team

Other (please specify)

Did you have a recent hospital appointment about the feeding tube? Yes/No

If yes, who did you see? (Tick all that apply)

Doctor

Dietitian

Feeding Therapy Team

Nurse

Speech Pathologist

Occupational Therapist

Other (please specify)

What information were you given about the feeding tube during the appointment?

Please indicate your agreement with the following statements (Strongly agree to Strongly disagree):

At the hospital appointment, I felt listened to.

At the hospital appointment, I felt supported.

At the hospital appointment, my questions were answered.

At the hospital appointment, my needs were met.

At the hospital appointment, I was given a plan for managing the feeding tube.

Is there anything else from the appointment you'd like to share?

Section 5: Reflections and Experiences

Is there any information you wish you had been told earlier about the feeding tube?

What has been your worst experience related to the feeding tube so far?

What has been your best experience related to the feeding tube so far?

Is there anything else you would like to share about your experience of living with the feeding tube?

File S2. Parent Interview Guide

Initial Interview Guide (Tube Insertion Phase)

1. When was the tube inserted?
2. What reason were you given for your child needing a feeding tube?
3. Who explained this to you (e.g., doctor, dietitian)?
4. Were you told in advance that your child would need a tube?
5. Were you involved in the decision to insert the feeding tube?
6. Who else was involved in the decision?
7. Were you involved in planning the feeding regimen?
8. Did you feel this was a joint decision or one made for you?
9. What is your understanding of how long your child will need the feeding tube?
10. Were you informed of any risks or benefits related to the feeding tube?
11. Were any alternatives to a feeding tube discussed?
12. Were you told about different types of feeding tubes?
13. Were you provided with information about the feeding tube? If yes, what was it (e.g., booklet, website)?
14. What was helpful about the information provided?
15. What could have been improved in the information provided?
16. Were you referred to anyone to support your child's eating or drinking?
17. When you go home, where do you think you will get information or advice about the feeding tube?
18. Would you like to speak to other families who have a child with a feeding tube?
19. Who do you think is the main person making decisions about your child's feeding tube?
20. What are the most important things for you and your child in this situation?
21. Who do you feel you can go to for support regarding the feeding tube?
22. Were you given any education about managing the feeding tube at home?
23. Were you taught how to insert the feeding tube?
24. Have you considered the cost of the feeding tube? Was this explained to you?
25. Do you have any other thoughts about the feeding tube you'd like to share?

Maintenance Interview Guide

1. Tell me about the experiences you are having with the feeding tube.

2. What is the impact of your child having a feeding tube?

3. Do you have any feedback on the care you have received regarding the feeding tube?

4. Did you feel able to talk to hospital clinicians about the feeding tube?

5. How was the communication?

6. Did you feel respected?

7. Did you feel your needs were met?

8. Was there any information you wish you had been told earlier?

9. What could be improved?

10. What support systems or networks do you have to help with the feeding tube?

11. Where are you getting information about the feeding tube?

12. What is your understanding of how long your child will need the feeding tube?

13. Have you been told about any side effects of the feeding tube?

14. Who do you think is the main person making decisions about your child’s feeding tube?

15. What are the important things for you and your child in this situation?

16. How has tube feeding affected your relationship with your child?

17. Are you receiving any feeding therapy for your child’s oral intake?

18. Do you have any other thoughts about the feeding tube you’d like to share?

<u>Final Interview Guide (Tube Removal / End Phase)</u>

1. Tell me about your overall experiences with the feeding tube.
2. What was the impact of your child having a feeding tube?
3. Do you have any feedback on the care your child received?
4. Did you feel able to talk to hospital clinicians about the feeding tube?
5. How was the communication?
6. Did you feel respected?
7. Did you feel your needs were met?
8. What information were you given about the tube?
9. What could be improved?
10. How did you troubleshoot issues with the feeding tube?
11. What support systems or networks did you have to help with the feeding tube?
12. Where did you get information about the feeding tube?
13. What was your understanding of the duration of tube feeding?
14. Who was the main person making decisions about your child's feeding tube?
15. Has tube feeding affected your relationship with your child?
16. What were the most important things for you and your child in this situation?
17. Was there any information you wish you had known earlier about tube feeding, and why?
18. What did you think of the financial cost of tube feeding?
19. Do you still have follow-up at the hospital? If yes, who are you seeing?
20. Did the feeding tube lessen or increase your worries about your child?
21. What was the hardest part of caring for your child with a feeding tube?
22. If you could change one thing to improve the healthcare system for children with feeding tubes, what would it be?
23. How confident did you feel navigating the healthcare system with your child's feeding tube?
24. What were your thoughts when the tube was removed? *(do not ask if the tube remains in place)*
25. Do you have any other thoughts about the feeding tube you'd like to share?

File S3. Caregiver Burden Questionnaire

This questionnaire was administered in Australia and all cost-related items were presented in Australian dollars (AUD). For publication, results in the main text have been converted to US dollars (USD) at a fixed rate of 0.6561 USD per AUD.

My child's feeding tube has been

- Not a financial burden at all
- a minor financial burden
- a moderate financial burden
- a significant financial burden
- a catastrophic financial burden
- Unsure
- General

Prior to your child needing a feeding tube, what was your yearly net income?

- Under $20,000
- $20,001 – $40,000
- $40,001 – $60,000
- $60,001 – $80,000
- $80,001 – $100,000
- $100,001 or over

What was your partner's yearly net income?

- Under $20,000
- $20,001 – $40,000
- $40,001 – $60,000
- $60,001 – $80,000
- $80,001 – $100,000
- $100,001 - $150,000
- Above $150,001
- Not applicable

What was your family's average annual income?

- Under $20,000
- $20,001 – $40,000
- $40,001 – $60,000
- $60,001 – $80,000
- $80,001 – $100,000
- $100,001 - $150,000
- Above $150,001

Did you own your current residence prior to your child needing a feeding tube? Yes/No

Before your child had a feeding tube, what type of work did you do?

- Government job
- Private company
- Individual contractor/freelancer
- Unemployed
- Student
- Other

Did you have to change your employment situation after your child needed a feeding tube?

- Unchanged
- Stopped working
- Short-term leave
- Long-term leave
- Reduced hours
- Turned down a promotion / raise in pay/ more work
- Not applicable

Was there a decrease in your salary after your child needed a feeding tube? If Yes – how much per month?

- Under $100
- $101 – $200
- $201 – $300
- $301 – $400
- $401 – $500
- $501 - $600
- $601 - $700
- Above $700
- No change

Before your child had a feeding tube, what type of work did your partner do?

- Government job
- Private company
- Self employed
- Unemployed
- Student
- Other
- Not applicable

Did your partner have to change their employment situation after your child had a feeding tube?

- Unchanged
- Stopped working
- Short-term leave
- Long-term leave
- Turned down a promotion / raise in pay/ more work
- Reduced hours
- Not applicable

Was there a decrease in their salary? If Yes – how much per month?

- Under $100
- $101 – $200
- $201 – $300
- $301 – $400
- $401 – $500
- $501 - $600
- $601 - $700
- Above $700
- No change
- Not applicable

Did you have any other friends or relatives that cared for your child after they needed a feeding tube?
Yes/No

If yes, did this impact on their ability to work? Yes/No

If yes, what was their monthly loss of income caring for your child?

- Under $100
- $101 – $200
- $201 – $300
- $301 – $400
- $401 – $500
- $501 - $600
- $601 - $700
- Above $700
- No change
- Not applicable

Financial support

Did you receive medical assistance for your child before they needed a feeding tube? Yes/No

Did you receive medical assistance for your child after they needed a feeding tube? Yes/No

Did you have health insurance for your child before they needed a feeding tube? Yes/No

Did you have health insurance for your child after they needed a feeding tube? Yes/No

Did you get any help with the medical expenses for your child? Yes/No

If yes – what help did you receive?

- reduced priced medications
- Home enteral nutrition script at a reduced cost
- NDIS (National Disability Insurance Scheme)
- Other

Did you get any help with the non-medical expenses for your child (e.g., transport, accommodation)? If yes – what help did you receive?

- Car parking vouchers
- Hospital accommodation
- Other
- Did you get any financial support from the government?
- If yes – what help did you receive?
- Carer's allowance
- Carer payment
- Carer supplement
- Child disability assistance
- Health care card
- Other

Did you get any non-governmental support? If yes – what help did you receive?

- Charity input
- Fund-raising
- Other
- Did you have any income protection - if you had to take leave from work?
- Yes
- No
- Not applicable

Direct Medical Costs

What was the total medical costs you paid relating to your child's feeding tube?

- Under $100
- $101 – $200
- $201 – $300
- $301 – $400
- $401 – $500
- $501 - $600
- $601 - $700

Did you have to pay any treatment charges related to your child's feeding tube (e.g., x-rays, GP fees, consultant fees) If yes – what were they and what were the costs?

- Under $100
- $101 – $200
- $201 – $300
- $301 – $400
- $401 – $500
- $501 - $600
- $601 - $700

Non- Direct Medical Costs

Did you have to buy any equipment for your child after they had a feeding tube? (e.g., feeding pump pole, new car seat). If yes, what did you need to buy and how much did it cost?

- Under $100
- $101 – $200
- $201 – $300
- $301 – $400
- $401 – $500
- $501 - $600
- $601 - $700

Did you need to hire help (e.g., babysitters/ carers) to help look after your child after they had their feeding tube? If yes, how much did this cost per month?

- Under $100
- $101 – $200
- $201 – $300
- $301 – $400
- $401 – $500
- $501 - $600
- $601 - $700

Did your family's communication increase after your child had a feeding tube? e.g., more phone use, internet? (beyond your usual expenses). If yes, how much did this cost per month?

- Under $100
- $101 – $200
- $201 – $300
- $301 – $400
- $401 – $500
- $501 - $600
- $601 - $700

After your child had a feeding tube, did you have to spent more money on hygiene (e.g. plastic gloves, laundry). If yes, how much did this cost per month?

- Under $100
- $101 – $200
- $201 – $300
- $301 – $400
- $401 – $500
- $501 - $600
- $601 - $700

Did you have to buy extra food (beyond your usual expenses) after your child had their feeding tube, (e.g., for your child, for hospital appointments, during the hospital admission for your child, for yourself, for your family). If yes what were the costs per month?

- Under $100
- $101 – $200
- $201 – $300
- $301 – $400
- $401 – $500
- $501 - $600
- $601 - $700

Did you have to buy extra clothes for your child after they had their feeding tube (beyond your usual expenses)? If yes – what were they, and what were the costs per month?

- Under $100
- $101 – $200
- $201 – $300
- $301 – $400
- $401 – $500
- $501 - $600
- $601 - $700

Did you have to buy gifts, treats, toys as feeding-tube related expenses for anyone in the family (including your child) after your child had a feeding tube? *For example promising to buy your other children a toy for sitting in the waiting room for a medical appointment related to your child's feeding tube.* **If yes – what were they, and what were the costs per month?**

- Under $100
- $101 – $200
- $201 – $300
-
- $301 – $400
- $401 – $500
- $501 - $600
- $601 - $700

Did you have to buy essential products during your hospital admission(s) (e.g., phone battery charger, pillows)? (beyond your usual expenses) If yes, what were they and what were the costs per month?

- Under $100
- $101 – $200
- $201 – $300
- $301 – $400
- $401 – $500
- $501 - $600
- $601 - $700

Did you need to get a cleaner after your child needed a feeding tube to help manage the workload at home? If yes, what was the cost per month?

- Under $100
- $101 – $200
- $201 – $300
- $301 – $400
- $401 – $500
- $501 - $600
- $601 - $700

Did you need to relocate your home after your child had their feeding tube, (e.g., to be closer to the hospital), If yes, what was the cost per month?

- Under $100
- $101 – $200
- $201 – $300
- $301 – $400
- $401 – $500
- $501 - $600
- $601 - $700

Were there any other expenses related to your child's feeding tube not mentioned above?

Hospital costs

What kind of transportation did you and your family usually take to and from the hospital when your child was an inpatient?

- Car
- Train
- Bus
- Taxi
- Other

How much money did you spent travelling to and from the hospital in total? Considering the cost of fuel for the car, car parking, train tickets, air travel, bus tickets, tolls on road?

- Under $100
- $101 – $200
- $201 – $300
- $301 – $400
- $401 – $500
- $501 - $600
- $601 - $700

How many times did you travel to the hospital when your child was an inpatient with their feeding tube?

- 0 - 5
- 6 - 10
- 11 - 20
- 21 - 30
- >50

Did you rent an apartment near the hospital for the convenience of your family during your child's hospitalisation? If yes, what was the cost per month?

- Under $100
- $101 – $200
- $201 – $300
- $301 – $400
- $401 – $500
- $501 - $600
- $601 - $700

Did you need to put your other children in childcare when your child was hospitalized? If yes, what was the cost per month?

- Under $100
- $101 – $200
- $201 – $300
- $301 – $400
- $401 – $500
- $501 - $600
- $601 - $700

Cost in time

How much more time did you need to spend looking after your child every day after they needed a feeding tube? For example giving tube feeds, cleaning the utensils, preparing the tube feeds. Approximately an extra..

- 1 hr. per day
- 2 hours per day
- 4 hours per day
- 6 hours per day
- 8 hours per day
- 10 hours per day
- 12 hours per day
- Other

How much time do you need to spend attending hospital/clinical/ therapy appointments related to your child's feeding tube? Approximately an extra..

- 1 hr. per week
- 2 hours per week
- 4 hours per week
- 6 hours per week
- 8 hours per week
- 10 hours per week
- 12 hours per week
- Other

How much time do you need to spend travelling to hospital/clinical/ therapy appointments related to your child's feeding tube? Approximately an extra

- 1 hr. per week
- 2 hours per week
- 4 hours per week
- 6 hours per week
- 8 hours per week
- 10 hours per week
- 12 hours per week
- Other

How much time do you need to spend communicating with hospital teams/ clinicians related to your child's feeding tube? Approximately an extra

- 1 hr. per week
- 2 hours per week
- 4 hours per week
- 6 hours per week
- 8 hours per week
- 10 hours per week
- 12 hours per week
- Other

Cost coping strategies

Have you had to…

- reduce your leisure activities like gym membership, eating out, movies
- reduce your spending on basic needs like food, electricity
- borrow money / used a credit card
- spread out clinician appointments
- use your savings
- sell any possessions
- ask your clinician for a cheaper prescription
- work more hours
- refuse a medical procedure because of the cost
- give your child less than their prescribed medication to make it last longer
- take out a second mortgage on your house

.. to help manage the cost of your child’s feeding tube.

Table S1. Participant Retention and Data Collected by Phase of Temporary Tube Feeding

Data collected	Parents* (n)	Children (n)	Notes
Recruitment	36	37	Includes one set of twins
			2 lost to follow-up immediately after recruitment
**Initial Phase**			
Interviews	29	30	5 excluded: established tube feeding >30 days
PedsQL	-	30	Same exclusion as interviews
Diary entries †	42 (28 parents)	-	
**Maintenance Phase**			
Interviews	24	25	5 early tube removals; 5 lost to follow-up
PedsQL	-	19	One child’s form not completed
Diary entries †	154 (27 parents)	-	
**Final Phase**			
Interviews	28	28	2 additional lost to follow-up
PedsQL	-	28	
Burden questionnaire	28	-	Parents only
Diary entries †	27 (27 parents)	-	

Note. * One parent completed interviews on behalf of the twins. At the first two timepoints, this was treated as a single interview, in line with the parent’s preference, as both children had similar experiences. For the final interview, one twin’s tube had been removed, so separate interviews were completed. † One parent didn’t complete any diary entries during their study duration

Table S2: Parent Quotes related to Financial Burden

Category	Subcategories	Representative Parent Quotes
Direct costs	• Tube supplies and equipment<br>• Private clinician costs<br>• Travel-related expenses	• *“No one has talked to me about that [the costs]”* [P06]<br>• *“We wish we had been prepared for what was involved, the cost”* [P19]<br>• *"We had to buy different tapes just for them to stick better... I’m retaping it 3-4 times a day*" [P05]<br>• *“We were running out of ph testing strips, I couldn't find them in 4 chemists, needed to order them online and that night he pulled the tube out”* [P13]<br>• *“We see a doctor who bulk bills us, so there's hasn't been any cost in that effect”* [P24]<br>• *“Our private paed, we were seeing her every three weeks and it was $280 [per] appointment*” [P26]<br>• *"I mean, the hospital car park, to be honest, is the big one... it's just such a question mark as to how much that's gonna cost you per week."* [P16]
Indirect costs	• Employment disruption<br>• Childcare challenges<br>• Time commitment	• *"Everything* [relating to tube feeding care] *got too much, so he* [partner] *quit his job - unemployed now"* [P21]<br>• *“I'm on leave without pay, [my partner] has reduced to half time to help at home, so our income has reduced considerably”* [P19]<br>• “*I don’t qualify to get carer’s payments, so I can’t stay home with her… I have to go back to work”* [P20]<br>• *“If she was .. to continue and stay on that [tube feeding] after as a toddler and I’m the one who needs to be able to operate that, that would definitely be a cost we'd have to factor in whether she could actually go to childcare, and me go back to work, or whether I need to be her carer kind of full time”* [P24]<br>• “*only the two of us at home can do it [tube feeds]. It's a real problem because it affects work for the two of us at home”* [P13]<br>• *“It took three attempts to get his new tube in this week, it wouldn't work and he was screaming and getting really worked up, it took a long time”* [P08].
Financial Support	• Government subsidies<br>• Information barriers and self-advocacy<br>• Support inadequacy	• *“We were just told that the nutrini [formula] will now be free, which is good news to us. It being free makes it less of a hassle”* [P10]<br>• *“She's covered under in the NDIS, so we don't have to pay anything”* [P18]<br>• *"I did find out just today actually that we were eligible to get the subsidy... I don't think we got told that at the start, but I just happened to ask today because they are quite expensive."* [P22]<br>• *“sometimes they're covered, sometimes they're not... and then all of a sudden it changes and you're paying $30 a tin* [for home enteral nutrition] to last six days” [P14]

Note: NDIS - National Disability Insurance Scheme, (a government-funded program providing support to those with disabilities).

Table S3. Reported Results in Australian Dollars (AUD)

Category	n	Mean (± SD)	Total Monthly Cost* (Mean ± SD)
**Annual Family Income**			
$0 – 60,000	7		
$60,001 – 100,000	6		
$100,000 and above	12		
Annual net family income	25	$62,500.00	
**Out-of-Pocket Direct Medical Costs**			**$898.53 ± 332.44**
Total medical costs related to feeding tube	25	$316.32 (± 275.40)	
Out of pocket costs for those that received the government subsidy	*11*	*$328.00 (± 263.00)*	
Out of pocket costs for those that did not receive the government subsidy	*13*	*$307.00 (± 295.00)*	
Treatment costs (e.g., GP fees)	22	$400.38 (± 284.92)	
Equipment for the tube	23	$128.57 (± 259.01)	
**Out-of-Pocket Direct Non-Medical Costs**			**$2277.32 ± 1002.27**
Hiring help (e.g., carers)	4	$225.25 (± 332.25)	
Communication (e.g., increased phone use)	13	$88.65 (± 269.79)	
Hygiene (e.g., extra laundry)	16	$137.78 (± 257.92)	
Buying extra food (e.g., for hospital visits)	20	$275.45 (± 201.98)	
Buying extra clothes	14	$128.79 (± 589.77)	
Hiring a cleaner	1	$50.00 (± 0.00)	
Total cost of travel to and from hospital †	24	$462.97 (± 262.37)	
**Inpatient Costs**			**$830.55 ± 332.44**
Extra essential products (e.g., pillows)	10	$179.68 (± 258.53)	
Accommodation for inpatient stay	4	$350.50 (± 23.36)	
Extra childcare during inpatient stay	4	$300.37 (± 207.69)	
**Outpatient Costs**			**$540.85 ± 505.68**
Extra gifts, treats, and toys	10	$180.30 (± 244.90)	
Essential outpatient products	5	$110.30 (± 295.06)	
Relocation (to be closer to hospital)	2	$250.25 (± 329.66)	

*Dollar amounts are AUD. † Travel was collected as a total over the study period, not per month, and is therefore presented separately and excluded from monthly non-medical subtotals.
